# Case Report: Intrahepatic cholestasis: a diagnostic dilemma

**DOI:** 10.12688/wellcomeopenres.19381.1

**Published:** 2023-05-24

**Authors:** Udit Acharya, Ram Chandra Panthi, Roshan Shrestha, Bimal Pandey, Sudeep Adhikari, Janak Koirala, Buddha Basnyat

**Affiliations:** 1 Internal Medicine, Patan Academy of Health Sciences, Lalitpur, Nepal

**Keywords:** Cholestasis, Intrahepatic cholestasis, BRIC

## Abstract

Cholestasis is an impairment of bile formation or bile flow. The mechanisms of cholestasis can be broadly classified into intrahepatic and extrahepatic. Most of the time, etiology can be determined with proper history, physical examination and diagnostic testing including laboratory and imaging tests. This is a case report of a patient with severe cholestasis who underwent extensive evaluation to determine the etiology of intrahepatic cholestasis.

This Case Report details a 28-year-old male who presented with seven days’ history of yellowish discoloration of eyes, passage of dark colored urine, generalized body itching, and passage of clay colored stool. Patient had no similar episode in the past. Laboratory investigations showed unconjugated hyperbilirubinemia and increased alkaline phosphatase. Magnetic resonance cholangiopancreaticography was unremarkable and liver biopsy was suggestive of cholestatic pattern and negative for acute hepatitis. The bilirubin level started decreasing after a month.

The etiology of intrahepatic cholestasis remained unknown. Genotyping could not be done due to limited available resources. It may have helped to diagnose familial hepatocellular cholestasis such as benign recurrent intrahepatic cholestasis (BRIC) and progressive familial intrahepatic cholestasis. Gamma glutamyl transferase is normal or mildly elevated in patients with BRIC. A similar picture was seen in our case as well. If the patient presents again with similar symptoms and findings, we can consider the diagnosis of BRIC.

## Introduction

Cholestasis is an impairment of bile formation or bile flow. Patients with cholestasis present with fatigue, pruritis and jaundice. Cholestasis may be intrahepatic or extrahepatic ^1^. The presentation of intrahepatic cholestasis and associated biochemical abnormalities may mimic biliary obstruction and can generate diagnostic confusion. Causes of intrahepatic cholestasis include primary biliary cirrhosis (PBC), primary sclerosing cholangitis (PSC), drugs and toxins, sepsis, malignancy, granulomatous liver disease, intrahepatic cholestasis of pregnancy, viral hepatitis, alcoholic hepatitis, genetic disorders, total parenteral nutrition associated cholestasis, graft versus host disease, post liver transplant cholestasis, etc ^2, 3^.

Clinically, cholestatic disorder manifests as jaundice and pruritus from the retention of bile acids in the blood with subsequent deposition in the skin, and symptoms secondary to malabsorption of fat and fat soluble vitamins ^4^. Biochemical markers of cholestasis include jaundice with predominant elevation of serum alkaline phosphatase (ALP) relative to aminotransfersases. Intrahepatic cholestatic disorders can be categorized histologically as infiltrative, those associated with injury to cholangiocytes within intrahepatic bile ductules, and those in which major histologic changes are not evident ^3^.

The diagnosis of intrahepatic cholestasis is made after imaging rules out extrahepatic biliary obstruction. . Depending on the clinical situation, evaluation requires different serological studies in order to determine etiology, and sometimes it needs to be confirmed with a liver biopsy ^5, 6^. Here we report a case of a patient who presented in a tertiary care hospital in Nepal in which the etiology of intrahepatic cholestasis remained unknown even after extensive workup.

## Case report

A 28-year-old male, Newar by ethnicity, from central Nepal and journalist by occupation, previously healthy, presented with seven days’ history of yellowish discoloration of eyes, passage of dark colored urine, generalized body itching, and passage of clay colored stool. The patient denied history of fever, abdominal pain, or viral prodrome. He used to consume alcohol on social occasions with last intake around one month prior to the illness. He did not have a history of intake of any medications including herbal supplements and had not previously had biliary tract surgery. There was no family history of jaundice or liver disease. Physical examination revealed icterus and excoriation marks all over the body. Other examination findings were unremarkable. Initial laboratory investigations revealed total bilirubin of 30.1 mg/dl (Reference: 0.4-1), direct bilirubin 19.3 mg/dl (Reference: 0.04-1), aspartate aminotransferase (AST) 14 units/liter (Reference: 0-37), alanine aminotransferase (ALT): 21.0 units/Liter (Reference: 5-35), alkaline phosphatase (ALP): 418 units/liter (Reference: 53-125), and gamma glutamyl transferase (GGT):17 units/liter (Reference: 8-61). Serum Albumin was 4.8 g/dl (Reference: 3-5), prothrombin time (PT): 14.7, international normalized ratio (INR): 1.05 (Reference: 1.0-1.3). Serological markers for hepatitis A, hepatitis B, hepatitis C, hepatitis E and human immunodeficiency virus (HIV) were negative.

**Figure 1.**
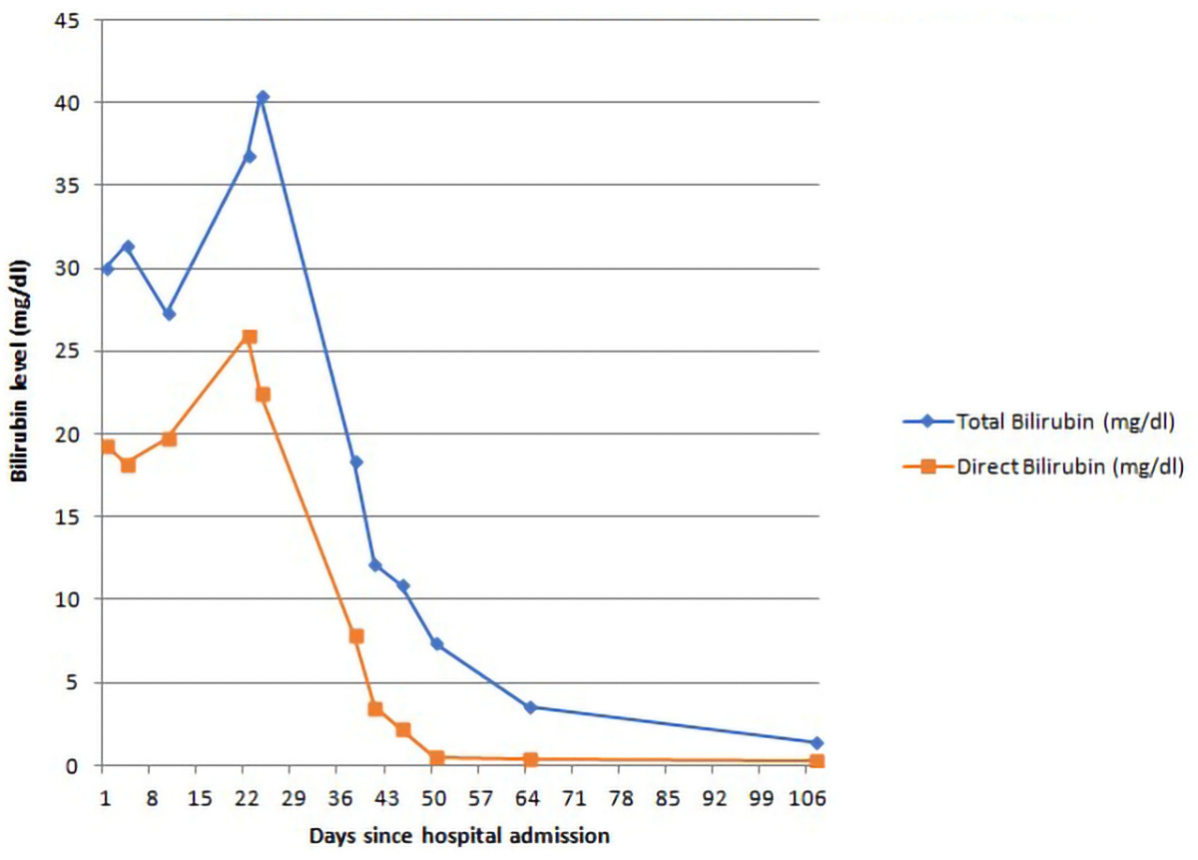
Trend in bilirubin level of the patient.

Patient was initially admitted in the surgical ward for evaluation of obstructive jaundice. Contrast enhanced computed tomography (CECT) of abdomen and magnetic resonance cholangiopancreaticography (MRCP) was done which did not show any abnormality in the biliary tract, pancreas, and peripancreatic region. After ruling out extrahepatic causes with imaging studies, the patient was shifted to the medical ward for further evaluation of intrahepatic cholestasis. Various investigations to determine the etiology of intrahepatic cholestasis were done including thyroid function tests, antinuclear antibody (ANA), liver kidney muscle-1 (LKM-1) antibody, anti-smooth muscle antibody (ASMA), antimitochondrial antibody (AMA), immunoglobulin G (IgG) -total, carcinoembryonic antigen (CEA), carbohydrate antigen 19.9 (CA-19.9), alpha fetoprotein (AFP), serum ceruloplasmin, 24-hour urinary copper and iron profile; the results of which did not reveal the etiology of intrahepatic cholestasis. Blood culture did not reveal any growth; serologies for brucellosis, leptospirosis and scrub typhus were all negative. Malaria antigen was negative. Patient was managed with ursodeoxycholic acid 300mg twice daily, cholestyramine 4g twice daily, levocetirizine 5mg once daily, fat soluble vitamins (cholicalciferol 60000 IU once, inj. vitamin K 10mg iv once), and other supportive measures such as IV fluids. The trend of bilirubin during his illness is shown in Figure 1.

During the hospital stay, he developed hospital-acquired pneumonia which was managed with inj. piperacillin-tazobactam 4.5g IV thrice daily for seven days, and the patient recovered from pneumonia in seven days. A liver biopsy was performed. The patient’s symptoms gradually improved, and his bilirubin also started to decrease gradually. So, he was discharged with advice to follow up with liver biopsy report. On follow up seven days after discharge, a histopathology report of liver biopsy showed bile stasis with minimal inflammatory cells infiltration, suggestive of cholestatic pattern and negative for acute hepatitis. However, etiology of intrahepatic cholestasis still remained unknown.

## Discussion

Intrahepatic cholestasis is considered once the extrahepatic cholestasis is ruled out by imaging modalities ^4^, as in our case in which CECT abdomen and MRCP were normal. On evaluation of etiology of intrahepatic cholestasis, our patient did not have a history of any drug intake or significant alcohol consumption. Serology for viral hepatitis and autoimmune hepatitis were negative. Tests results were also negative for primary biliary cirrhosis and primary sclerosing cholangitis as AMA and MRCP were normal, respectively. Liver biopsy ruled out small duct PSC. Serum ceruloplasmin, slit-lamp exam for Kayser Fleischer ring, and 24-hour urinary copper were negative for Wilson`s disease. Iron studies did not suggest hemochromatosis as the cause for intrahepatic cholestasis. Genetic testing should also be considered such as mutations in the hepatobiliary transporters ATP8B1, ABCB11 and ABCB4 in the evaluation of familial hepatocellular cholestasis like progressive familial intrahepatic cholestasis (PFIC) or benign recurrent intrahepatic cholestasis (BRIC). However, genetic testing was not done in our case because of the limited available resources.

Progressive familial intrahepatic cholestasis (PFIC) is a heterogeneous group of disorders, characterized by defective secretion of bile acids or other components of bile. PFIC is unlikely in our case as these disorders usually present during infancy or childhood, and are associated with growth failure and progressive liver disease.

Benign recurrent intrahepatic cholestasis (BRIC) is characterized by recurrent cholestatic episodes. Patients present with this disease for the first time at a young age, usually infancy to late adulthood. The frequency of attacks can vary, ranging from once in a few months to once in a decade. During the attacks, the patients present with malaise, anorexia, pruritus, weight loss, jaundice and malabsorption. Laboratory tests reveal biochemical evidence of cholestasis (conjugated hyperbirubinemia, raised ALP). GGT is normal or mildly elevated in patients with BRIC. A similar picture was seen in our case as well.

A few factors made the diagnosis of BRIC challenging in our case. Although BRIC can present for the first time in adulthood, it usually presents before the age of 20, and our patient was nearly 30. Second, BRIC is a recurring disease. The patient had no similar episode in the past. The diagnosis of BRIC could not be ascertained due to it being the first presentation.

Liver biopsy is important in workup of patients with cholestatic jaundice who have a normal imaging and no other obvious etiology ^1^. However, it may not be adequately sensitive, and may have complications such as bleeding. Although histological examination of liver tissue confirmed the diagnosis of intrahepatic cholestasis, the cause still remained unanswered in this case.

If the patient presents again with similar symptoms and findings, we can consider the diagnosis of BRIC. Genotyping would be helpful to reach the diagnosis.

## Informed consent

Written informed consent was taken from patient before submission.

## Data Availability

All data underlying the results are available as part of the article and no additional source data are required.
