# Supplementary material for: Case Report: Intrahepatic cholestasis: a diagnostic dilemma
Source: Wellcome Open Res. 2024 Mar 21;8:226. Originally published 2023 May 24. [Version 2] doi: 10.12688/wellcomeopenres.19381.2 (PMC11589421; doi:10.12688/wellcomeopenres.19381.2)
Supplement: Peer review history

# Reviewer report: Reviewer response for version 2

**DOI:** 10.21956/wellcomeopenres.23554.r110997

**Published:** 2024-11-25

**Authors:** Bagus Setyoboedi, Rendi Aji Prihaningtyas

**Affiliations:** 1 Universitas Airlangga, Surabaya, Indonesia; 2 Child Health, Universitas Airlangga (Ringgold ID: 148005), Surabaya, East Java, Indonesia

This case report has a clear backgdound. The case is described in detail and a detailed examination has been conducted. Application in clinical practice is easy but requires costly investigations.Liver function test charts such as AST and ALT can be included as well as GGT evaluation to further reveal the trend of treatment success.

Are enough details provided of any physical examination and diagnostic tests, treatment given and outcomes?

Yes

Is the case presented with sufficient detail to be useful for other practitioners?

Yes

Is sufficient discussion included of the importance of the findings and their relevance to future understanding of disease processes, diagnosis or treatment?

Yes

Is the background of the case’s history and progression described in sufficient detail?

Yes

Reviewer Expertise:

hepatology

We confirm that we have read this submission and believe that we have an appropriate level of expertise to confirm that it is of an acceptable scientific standard.

# Reviewer report: Reviewer response for version 2

**DOI:** 10.21956/wellcomeopenres.23554.r92307

**Published:** 2024-08-23

**Authors:** Tai Ren, Yang Li

**Affiliations:** 1 Shanghai Jiao Tong University, Shanghai, China; 2 Department of Biliary-Pancreatic Surgery, Renji Hospital, Shanghai, China

The authors reported an interesting case of severe intrahepatic cholestasis. The laboratory and imaging results provide logical and necessary information to exclude a extrahepatic source. The comprehensive serological results did not support other liver diseases but indicate a possible genetic origin. In addition to the trends in TB, DB, and ALP, it would be of interest to show the trend in GGT during the process. Though a confirmative genetic assessment was not performed, I agree that BRIC is likely to be the diagnosis. The case is in general well-presented. Some language issues may require further revision (eg. two verbs in "The diagnosis of intrahepatic cholestasis is made after imaging rules out extrahepatic biliary obstruction.").

Are enough details provided of any physical examination and diagnostic tests, treatment given and outcomes?

Yes

Is the case presented with sufficient detail to be useful for other practitioners?

Partly

Is sufficient discussion included of the importance of the findings and their relevance to future understanding of disease processes, diagnosis or treatment?

Partly

Is the background of the case’s history and progression described in sufficient detail?

Yes

Reviewer Expertise:

Hepatobiliary diseases, perinatal care, epidemiology

We confirm that we have read this submission and believe that we have an appropriate level of expertise to confirm that it is of an acceptable scientific standard, however we have significant reservations, as outlined above.

# Reviewer report: Reviewer response for version 1

**DOI:** 10.21956/wellcomeopenres.21468.r69965

**Published:** 2023-12-04

**Authors:** Kate Lynch

**Affiliations:** 1 Royal Adelaide Hospital Adelaide, Adelaide, Australia

It would have been useful to know if the serologies mentioned were completely normal or just not "confirming" a disease. ANCA may have also been useful in the setting of small duct-PSC, where it can be patchy.

 The normal GGT with raised bilirubin and ALP, and the lack of abdominal pain, should be brought out strongly. When one sees a normal GGT with cholestasis, one automatically thinks of the PFIC and BRIC disorders. 

 I disagree that the patient's age of presentation makes the diagnosis confusing. I often diagnose it in early adulthood. It may not have been evident previously as was subclinical, where this was a severe flare. Were there any prior liver function tests available to evaluate?

 I agree the most likely diagnosis is BRIC. We see this more commonly in women when they start using hormonal therapy and/or become pregnant, and cases can be severe like this. Option can also include nasobiliary drainage if symptoms not responding to UDCA/colestyramine/rifampicin.

 Interesting case though.

Are enough details provided of any physical examination and diagnostic tests, treatment given and outcomes?

Yes

Is the case presented with sufficient detail to be useful for other practitioners?

Yes

Is sufficient discussion included of the importance of the findings and their relevance to future understanding of disease processes, diagnosis or treatment?

Partly

Is the background of the case’s history and progression described in sufficient detail?

Yes

Reviewer Expertise:

Autoimmune liver disease, cholestatic liver diseases, inflammatory bowel disease

I confirm that I have read this submission and believe that I have an appropriate level of expertise to confirm that it is of an acceptable scientific standard, however I have significant reservations, as outlined above.

# Response

**Published:** 2024-03-16

**Authors:** Sudeep Adhikari

**Affiliations:** Patan Academy of Health Sciences, Nepal

Thank you for the comments. Below is the detailed point by point response to your comments.

 Q- It would have been useful to know if the serologies mentioned were completely normal or just not "confirming" a disease. ANCA may have also been useful in the setting of small duct-PSC, where it can be patchy.

 A- ANCA was not done in our case. However other serologies mentioned in the manuscript were completely normal.

 Q- The normal GGT with raised bilirubin and ALP, and the lack of abdominal pain, should be brought out strongly. When one sees a normal GGT with cholestasis, one automatically thinks of the PFIC and BRIC disorders. 

 A- Thank you for the comment.

 Q- I disagree that the patient's age of presentation makes the diagnosis confusing. I often diagnose it in early adulthood. It may not have been evident previously as was subclinical, where this was a severe flare. Were there any prior liver function tests available to evaluate? A- We agree with your comment. Changes have been made in the manuscript. There was no prior LFT done in this case.

 Q- I agree the most likely diagnosis is BRIC. We see this more commonly in women when they start using hormonal therapy and/or become pregnant, and cases can be severe like this. Option can also include nasobiliary drainage if symptoms not responding to UDCA/colestyramine/rifampicin. Interesting case though.

 A- Thank you for the comment.

# Reviewer report: Reviewer response for version 1

**DOI:** 10.21956/wellcomeopenres.21468.r69971

**Published:** 2023-12-04

**Authors:** Holmfridur Helgadottir

**Affiliations:** 1 Haraldsplass Deaconal Hospital (HDS), Bergen, Norway

This case of severe intrahepatic cholestasis is interesting, the absence of abnormality on imaging and exclusion of other causes of liver disease is important when considering genetic liver disease. When the laboratory tests show profound cholestasis and normal GGT that raises suspicion for BRIC and other forms of familial intrahepatic cholestasis. Normal or minimally elevated GGT is the hallmark of BRIC. However, in this case, although the patient had extensive evaluation the genetic test is missing, and when that is not done the patient needs to have at least two attacks of jaundice separated by a symptom free interval to meet the diagnostic criteria.  

 We have > 100 BRIC cases reported worldwide, could this be the first case in Nepal?

**Introduction: **

- It is not correct to use bile acids in this sentence/statement: *“Clinically, cholestatic disorder manifests as jaundice and pruritus from the retention of bile acids in the blood…” *
  The jaundice is a manifestation of hyperbilirubinemia and the pathogenesis of pruritus in cholestatic disorders is complex; various candidate pruritogens have been studied and bile acids is just one of many.
- Jaundice is also not a biochemical marker, it is a clinical manifestation of cholestasis.
- One sentence has two period marks: “ *out extrahepatic biliary obstruction. .”*

**Case report: **

- Ref. values are provided for laboratory results, except for prothrombin time.
- When was CT taken, at admission or later (please provide day number during admission)? CT scan may identify gallstones but it's usually not as effective as the ultrasound – in this case it is important to rule out gallstones that could have passed through (important ddx).
- Was there not done abdominal ultrasound during admission to the hospital?
- Was MRI done early in admission (please provide day number during admission)?
- How was medical treatment chosen? What was the indication for the treatment of choice?
  Was pruritus’ the indication for medical treatment? And can you provide any information on the effect the medications had on the symptom?
- Can you provide UDCA dose in mg/kg for the case?
- What was the indication for K vitamin injection, was there rise in INR value during hospital stay?
- The liver biopsy, was the histology demonstrating centrilobular cholestasis?

**Figure 1:**

- The figure only shows bilirubin and DB. It would be interesting to also show the changes/curve for ALP.
- Did GGT stay normal the whole time?
- Was there any change in ALT and AST – that is sometimes seen in BRIC attacks.

**Discussion:**

- It is true that this could be a BRIC attack, however PFIC is very unlikely. PFIC typically presents in early life, rarely in early adulthood – this section can be shortened in the discussion.
- BRIC does not usually present in infancy to late adulthood – in most cases the first attack occurs during the patient’s teens or twenties. This should be corrected. The diagnosis has been made in infancy in families with a history of BRIC.
- Here the attack lasted a month and also bilirubin started decreasing after a month. How was the duration of the attack counted and how long was the admission time (hospital stay)?
- How long was the duration until all laboratory results were normal? In the literature the mean duration is approximately 12 weeks. I would also recommend to count the duration from the first sign/symptom.
- The attacks can have complications, such as pancreatitis or kidney failure, was there elevation of amylase, lipase or creatinine?
- The treatment choices for BRIC attacks should be mentioned in discussion.  
- The etiology of the case remained unknown – the diagnosis of BRIC can be confirmed by genetic testing after just one attack and that should be stated as the gold standard in the discussion. Before the genetic testing the diagnosis was more difficult, and patients had to have a minimum of two attacks of jaundice separated by intervals of weeks to years without any symptoms or cholestasis.
- I would recommend to get genetic testing, which is the gold standard for diagnosis as well as for the classification of the disease. This could also be of importance to decide for further follow-up and the choice of treatment of the next attack.

Are enough details provided of any physical examination and diagnostic tests, treatment given and outcomes?

Yes

Is the case presented with sufficient detail to be useful for other practitioners?

Partly

Is sufficient discussion included of the importance of the findings and their relevance to future understanding of disease processes, diagnosis or treatment?

Partly

Is the background of the case’s history and progression described in sufficient detail?

Yes

Reviewer Expertise:

Cholestatic liver diseases and cholestatic pruritus

I confirm that I have read this submission and believe that I have an appropriate level of expertise to confirm that it is of an acceptable scientific standard, however I have significant reservations, as outlined above.

# Response

**Published:** 2024-03-16

**Authors:** Sudeep Adhikari

**Affiliations:** Patan Academy of Health Sciences, Nepal

Thank you for your comments. Below is the detailed point by point response to your comments.

 Introduction:

 Q- It is not correct to use bile acids in this sentence/statement: “Clinically, cholestatic disorder manifests as jaundice and pruritus from the retention of bile acids in the blood…”

 A- Suggested changes have been made in the manuscript.

 Q- The jaundice is a manifestation of hyperbilirubinemia and the pathogenesis of pruritus in cholestatic disorders is complex; various candidate pruritogens have been studied and bile acids is just one of many.  

 A- Suggested changes have been made in the manuscript.

 Q- Jaundice is also not a biochemical marker, it is a clinical manifestation of cholestasis.  

 A- Suggested changes have been made in the manuscript.

 Q- One sentence has two period marks: “out extrahepatic biliary obstruction. .”

 A- Suggested changes have been made in the manuscript.  

 Case report:

 Q- Ref. values are provided for laboratory results, except for prothrombin time.  

 A- Suggested changes have been made in the manuscript.

 Q- When was CT taken, at admission or later (please provide day number during admission)? CT scan may identify gallstones but it's usually not as effective as the ultrasound – in this case it is important to rule out gallstones that could have passed through (important ddx). Was there not done abdominal ultrasound during admission to the hospital? Was MRI done early in admission (please provide day number during admission)?  

 A- CT scan was done on day 3, MRCP on day 4. Ultrasound of the abdomen was done soon after admission which showed no abnormality.

 Q- How was medical treatment chosen? What was the indication for the treatment of choice? Was pruritus’ the indication for medical treatment? And can you provide any information on the effect the medications had on the symptom?

 A- Medical treatment was given to relieve patient of pruritus. After the treatment, pruritus was improved. Jaundice was also reduced.  

 Q- Can you provide UDCA dose in mg/kg for the case?  

 A- UDCA was given at the dose of 15 mg/kg/day.

 Q- What was the indication for K vitamin injection, was there rise in INR value during hospital stay?  

 A- Vitamin K injection was given as empiric treatment for possible reduced fat soluble vitamin level due to cholestasis. However, INR was normal during hospital stay.

 Q- The liver biopsy, was the histology demonstrating centrilobular cholestasis?

 A- There was no centrilobular cholestasis seen in biopsy.

 Q- Figure 1: The figure only shows bilirubin and DB. It would be interesting to also show the changes/curve for ALP.

 A- ALP trend figure has been added. 

 Q- Did GGT stay normal the whole time?  

 A- GGT was normal.

 Q- Was there any change in ALT and AST – that is sometimes seen in BRIC attacks.

 A- There was slight increment in values of ALT and AST. Discussion:

 Q- It is true that this could be a BRIC attack, however PFIC is very unlikely. PFIC typically presents in early life, rarely in early adulthood – this section can be shortened in the discussion.

 A- Suggested changes have been made in the manuscript.  

 Q- BRIC does not usually present in infancy to late adulthood – in most cases the first attack occurs during the patient’s teens or twenties. This should be corrected. The diagnosis has been made in infancy in families with a history of BRIC.

 A- Suggested changes have been made in the manuscript.  

 Q- Here the attack lasted a month and also bilirubin started decreasing after a month. How was the duration of the attack counted and how long was the admission time (hospital stay)? A- Hospital stay was 34 days.

 Q- How long was the duration until all laboratory results were normal? In the literature the mean duration is approximately 12 weeks. I would also recommend to count the duration from the first sign/symptom.

 A- His bilirubin normalized in about 8 weeks and ALP normalized in about 16 weeks after the onset of illness. 

 Q- The attacks can have complications, such as pancreatitis or kidney failure, was there elevation of amylase, lipase or creatinine?  

 A- He had developed AKI during hospital stay, but no pancreatitis.

 Q- The treatment choices for BRIC attacks should be mentioned in discussion.    

 A- Suggested changes have been made in the manuscript.

 Q- The etiology of the case remained unknown – the diagnosis of BRIC can be confirmed by genetic testing after just one attack and that should be stated as the gold standard in the discussion. Before the genetic testing the diagnosis was more difficult, and patients had to have a minimum of two attacks of jaundice separated by intervals of weeks to years without any symptoms or cholestasis.

 A- Suggested changes have been made in the manuscript.

 Q- I would recommend to get genetic testing, which is the gold standard for diagnosis as well as for the classification of the disease. This could also be of importance to decide for further follow-up and the choice of treatment of the next attack.

 A- Genetic testing is not available in our setup so could not be done. It has been acknowledged in the manuscript.
